# Mechanical Power and Ventilator Variables in Patients With Acute Respiratory Distress Syndrome: A Pilot Prospective Cohort

**DOI:** 10.7759/cureus.111719

**Published:** 2026-06-29

**Authors:** Yashita Joshi, Sumit Kumar, Madhur Gairola

**Affiliations:** 1 Critical Care Medicine, Employees’ State Insurance Corporation Model Hospital and Pandit Bhagwat Dayal Sharma Post Graduate Institute of Medical Sciences, Rohtak, Faridabad, IND; 2 General Surgery, Vardhman Mahavir Medical College and Safdarjung Hospital, New Delhi, IND

**Keywords:** acute respiratory distress syndrome (ards), computed tomography (ct), lung compliance, mechanical ventilation, ventilator-induced lung injury

## Abstract

Background: Mechanical power (MP) integrates multiple ventilatory variables and reflects the total energy transferred to the lungs during mechanical ventilation, potentially contributing to ventilator-induced lung injury (VILI) in acute respiratory distress syndrome (ARDS). This study evaluated the association between MP, ventilatory variables, and mortality in patients with ARDS.

Methods: In this prospective cohort study conducted between May 2025 and May 2026, 50 mechanically ventilated patients with ARDS were enrolled and categorized into survivors (n=25) and non-survivors (n=25). Ventilatory parameters, arterial blood gas (ABG) variables, and MP components were serially recorded. Chest computed tomography (CT) was performed for quantitative lung aeration analysis. Mechanical power was calculated using static, dynamic, and resistive components.

Results: Non-survivors had significantly higher Acute Physiology and Chronic Health Evaluation II (APACHE II) and Sequential Organ Failure Assessment (SOFA) scores (p<0.0001). They demonstrated worse oxygenation, higher partial pressure of carbon dioxide (PaCO₂) and lactate levels, lower compliance, and significantly elevated ventilatory pressures and MP indices. Day 1 total MP, dynamic MP, and resistive MP were significantly greater among non-survivors (p<0.0001). Average ventilatory parameters during ICU stay showed persistent elevation of respiratory rate (RR), plateau pressure (PP), driving pressure (DP), and total MP in non-survivors. CT-derived aeration parameters did not differ significantly between groups. Multivariate regression identified average pressure of arterial oxygen (PaO₂), PaO₂/fraction of inspired oxygen (FiO₂) (P/F) ratio, day 1 static compliance (SC), plateau pressure, static MP, average peak inspiratory pressure (PIP), and average total MP as independent predictors of mortality.

Conclusion: Increased mechanical power and adverse ventilatory mechanics were strongly associated with mortality in ARDS. Continuous optimization of ventilatory parameters and reduction of cumulative mechanical energy delivery may improve outcomes in mechanically ventilated patients with ARDS.

## Introduction

Acute respiratory distress syndrome (ARDS) is a life-threatening respiratory condition characterized by severely impaired oxygen exchange due to damage to the alveolar-capillary barrier, which causes the lungs to become stiff and noncompliant. One of the hallmark features of ARDS is the patchy or uneven distribution of lung involvement, typically with the lower lobes sustaining more damage than the upper ones, leading to region-specific reductions in compliance. This heterogeneous pattern of lung injury causes variable responses to respiratory support measures [1]. Despite recent advances, it continues to be a major cause of mortality among critically ill patients, with death rates consistently ranging between 30% and 40%, with approximately 11.4% of patients needing mechanical ventilation [2].

Lung-protective ventilation in the treatment of ARDS focuses on minimizing harm by employing reduced tidal volumes (VT), limiting driving pressure (ΔP) and plateau pressure (PP), maintaining controlled respiratory rates (RR), and applying sufficient levels of end-expiratory pressure. Nevertheless, it remains unclear which individual ventilatory components most strongly correlate with better prognosis in ARDS [3].

In 2016, Gattinoni et al. proposed a unified concept of mechanical power (MP), which measures the total energy imparted to lung structures over time by incorporating multiple ventilatory variables, thereby elucidating how each contributes to the progression of ventilator-associated lung damage. This cumulative energy is believed to play a key role in the pathogenesis of ventilator-induced lung injury (VILI) [4].

Gattinoni et al. formulated a simplified method to calculate MP by utilizing the classical equation of motion. This method quantifies the total energy transferred to the lungs during mechanical ventilation by capturing three essential components: elastic static, elastic dynamic, and resistive forces. The elastic static element reflects the energy exerted through positive end-expiratory pressure (PEEP), while the elastic dynamic portion accounts for the energy required to stretch the lung’s elastic tissues during each tidal breath. The resistive component encompasses the energy expended to overcome airway resistance and maintain airflow throughout the respiratory cycle [4]. It was highlighted that MP, while important in understanding VILI, does not solely account for the extent of injury. Various studies have emphasized that the spatial distribution and the specific contributions of each ventilatory component to MP play a critical role in determining lung injury severity, even when total MP values are similar [5].

The primary aim of this study was to study any association of mechanical power between survivors and non-survivors of ARDS. The secondary aim was to study any correlation of mechanical power with primary ventilator variables, such as VT, DP, RR, and the amount of aerated lung tissue among survivors and non-survivors.

## Materials and methods

Study design and period

This pilot prospective cohort study was carried out at a tertiary care teaching hospital between May 21, 2025, and May 21, 2026, to identify the role of mechanical power and ventilator variables in patients with acute respiratory distress syndrome.

Ethical approval

Approval was received from the institutional ethics committee of ESIC Medical College and Hospital (vide approval number: 134X/11/13/2025-IEC/DHR/27, dated May 20, 2025), and the study was registered with the Clinical Trials Registry - India (vide registration number: CTRI/2025/07/091172). Informed and written consent was given by all the patients’ caretakers for their participation in the trial and for the publication of their data for academic purposes. The study was carried out in accordance with the principles of the Declaration of Helsinki 2013.

Patient selection

Patients in the age group of 18-65 years presenting with ARDS (as per the New Global Definition) on invasive volume control mechanical ventilation were recruited for the study. Exclusion criteria included patients with spontaneous breathing efforts, patients requiring pressure control mode ventilation, death on the day of admission, history of chronic obstructive or restrictive lung disease, and patients with chest wall injuries, pregnancy, and obesity (body mass index: >35 kg/m^2^).

Data collection and measurements

Patients who presented in the Department of Critical Care Medicine with ARDS who met the eligibility criteria were recruited for the study. ARDS was defined as per the New Global Definition of ARDS 2023 and all intubated patients were classified as mild (200 mmHg <PaO₂:FiO₂ ratio ≤ 300 mmHg or 235 < SpO₂:FiO₂ ≤ 315 (if SpO₂ ≤ 97%)), moderate (100 mmHg PaO₂:FiO₂ ratio ≤ 200 mmHg or 148 < SpO₂:FiO₂ ≤ 235 (if SpO₂ ≤ 97%)), or severe (PaO₂:FiO₂ ratio ≤ 100 mmHg or SpO₂:FiO₂ ≤ 148 (if SpO₂ ≤ 97%)) [6].

ICU admission date was considered as day 0. The next date of ICU admission was defined as day 1. The following patient data were recorded within 24 hours after admission: age, gender, body mass index, medical history, and Sequential Organ Failure Assessment (SOFA) [7] and Acute Physiology and Chronic Health Evaluation II (APACHE II) [8] scores. On arrival in the ICU, arterial blood gas was taken, and the PaO₂/FiO₂ ratio was noted. Patients were followed until the study endpoint, and then, retrospectively, they were grouped under survivors and non-survivors for further analysis.

Ventilator settings

At baseline, patients were maintained deeply sedated and paralyzed, ventilated in volume control with a square wave form without any inspiratory pause, applying a tidal volume between 4 and 8 mL/kg of 12 predicted body weight with a positive end-expiratory pressure (PEEP) value set by the attending physician to ensure an arterial saturation between 88% and 95%. Ventilator settings were adjusted on the basis of arterial blood gas values after one hour of the first setting. Ventilator setup and adjustment were performed at regular intervals of four hours and as necessary, based on the ARDS Clinical Network Mechanical Ventilation Protocol [9].

Baseline endotracheal aspirate for gram staining and culture sensitivity was sent on intubation.

Blood values of arterial pH, partial pressure of arterial oxygen (PaO₂), partial pressure of arterial carbon dioxide (PaCO₂), ratio of PaO₂ and fraction of inspired oxygen (FiO₂) (PaO₂/FiO₂), were recorded on arrival in the ICU and at an interval of eight hours subsequently. Mechanical ventilator settings, which included VT (scaled to the predicted body weight (PBW)), RR, PEEP, respiratory system compliance, elastance, plateau pressure, and driving Pressure (DP), were recorded on intubation and then at an interval of four hours subsequently. Serial data was recorded fourth hourly for a maximum of 14 days. The removal of the neuromuscular blocker or death was determined the end of the data collection.

Chest radiography

A computed tomography (CT) scan of the chest was taken after 24-48 hours of intubation after initial stabilization of the patient. A scan was performed in the inspiratory phase with PEEP of 5 cm H2O in all patients for quantitative analysis of lung volume, sustained by a portable ventilator (Bellavista 1000 Ventilator, Vyaire, Mettawa, IL). A multidetector row 13 computed tomography (Philips Ingenuity 128 slice CT Scanner, Philips, Netherlands) from the thoracic inlet to the diaphragm was performed. All images were reconstructed using standard reconstruction algorithms with a slice thickness of 1.0 mm and a reconstruction interval of 0.8 mm. The lung gas volume and amount of well-inflated tissue were computed with an automated algorithm software (Philips Intellispace portal 11 solutions). Lung regions were classified into four categories by CT attenuation densities: (1) hyperinflated, density between -1000 and -901 Hounsfield units (HU); (2) normally aerated, density between -900 and -501 HU; (3) poorly aerated, density between -500 and -101 HU; and (4) non-aerated, density between +100 and -100 HU.

Mechanical power was obtained as the algebraic sum of three components [10]: (1) elastic-static power (J/min) = 0.098 *VT*RR*PEEP; (2) elastic-dynamic power (J/min) = 0.098*VT*RR*0.5*DP; and (3) resistive power (J/min) = 0.098 * VT * RR * (Ppeak-Pplat).

Statistical analysis

The sample size for the comparison of mechanical power among survivors versus non-survivors was calculated based on the study by Coppola et al., and the mean mechanical power among survivors was 14.97 J/min. Assuming a 35% higher mechanical power among non-survivors will lead to 20.20 J/min [11]. Thus, the minimum sample size required in each group was calculated as 23, and taking a non-response rate of 10%, it came to be 25 in each group. The data were compiled and analyzed using MS Excel Office 365 (Microsoft Corp., Redmond, WA), GraphPad Prism 8.4.2 (Dotmatics, Boston, MA), and SPSS version 25 (IBM Corp., Armonk, NY). Descriptive statistics were presented in the form of proportions/percentages for categorical variables and median/interquartile range, along with mean and standard deviation (SD) for continuous data. Fisher’s exact test/chi-square test was used for the comparison of proportions (categorical variables). Continuous variables were analyzed using the Mann-Whitney test (independent group/unpaired data) and the Wilcoxon signed-rank test (for paired data). Univariate and multivariate analyses were done using Spearman correlation for non-parametric dependent data variables and regression (for bivariate dependent variables) models. Standardized and unstandardized beta covariates were estimated. A p-value of <0.05 was considered significant.

## Results

A total of 50 patients with ARDS were included in the study and were followed until the study endpoint. They were then categorized into survivors (n=25) and non-survivors (n=25). Baseline demographic characteristics, including age, gender, and BMI, were comparable between the groups. However, disease severity scores were significantly higher among non-survivors. Mean APACHE II score was 24.56±8.19 in non-survivors versus 13.84±5.88 in survivors (p<0.0001), while mean SOFA score was 11.12±4.04 versus 6.04±2.37 (p<0.0001), respectively (Table 1).

**Table 1:** Demographic and clinical parameter comparison Data is presented as mean±SD or number (%). Comparisons between survivors and non-survivors were performed using Student’s t-test for continuous variables and Pearson’s chi-square test for categorical variables. Statistical significance was defined as p<0.05. BMI: body mass index, APACHE II: Acute Physiology and Chronic Health Evaluation II, SOFA: Sequential Organ Failure Assessment, SD: standard deviation

Parameters	Non-survivor	Survivor	P-value	Test statistic
Age (in years)				Student’s t-test
Mean±SD	45.36±12	42.76±13.05	0.461	t=0.733
Gender				Chi-square test
Female	13 (52%)	15 (60%)	0.776	x^2^=0.325
Male	12 (48%)	10 (40%)		
BMI				Student’s t-test
Mean±SD	23.25±3.16	23.94±2.19	0.405	t=0.897
APACHE II score				Student’s t-test
Mean±SD	24.56±8.19	13.84±5.88	<0.0001	t=5.316
SOFA score				Student’s t-test
Mean±SD	11.12±4.04	6.04±2.37	<0.0001	t=5.423
Disease severity				Chi-square test
Moderate	0	3 (12%)	0.234	x^2^=3.191
Severe	25 (100%)	22 (88%)		

Pulmonary ARDS was more common, accounting for 72% of cases, whereas extrapulmonary involvement was seen in 28% of patients. Sepsis was the most frequent etiology of ARDS (16%), followed by H1N1 infection (14%), *Klebsiella pneumoniae* infection (12%), and carbapenem-resistant *Acinetobacter baumannii* (CRAB) and *Pseudomonas* infections (10% each).

Arterial blood gas analysis

Day 1 ABG analysis demonstrated significantly worse oxygenation and acid-base status among non-survivors. Mean pH was significantly lower in non-survivors (7.22±0.05 versus 7.39±0.03, p<0.0001), while PaCO₂ was markedly higher (55.06±5.54 versus 40.01±3.64 mmHg, p<0.0001). PaO₂ and P/F ratio were significantly reduced in non-survivors (53.13±6.32 mmHg and 66.41±7.90, respectively) compared with survivors (78.64±7.46 mmHg and 156.47±14.45, respectively; p<0.0001 for both). Lactate levels were significantly elevated among non-survivors (3.56±0.64 versus 1.78±0.26 mmol/L, p<0.0001). Similar trends persisted in average ABG values throughout ICU stay (Table 2).

**Table 2:** Arterial blood gas findings Data are presented as mean±SD. Group comparisons were performed using Student’s t-test. Statistical significance was defined as p<0.05. ABG: arterial blood gas, PaCO₂: partial pressure of carbon dioxide, PaO₂: partial pressure of oxygen, P/F ratio: PaO₂/FiO₂, SD: standard deviation

Parameters	Non-survivor	Survivor	P-value	Test statistic
Day 1 ABG				
Ph	7.22±0.05	7.39±0.03	<0.0001	14.36
PaCO₂	55.06±5.54	40.01±3.64	<0.0001	11.33
PaO₂	53.13±6.32	78.64±7.46	<0.0001	13.1
P/F ratio	66.41±7.90	156.47±14.45	<0.0001	27.54
Lactate	3.56±0.64	1.78±0.26	<0.0001	12.99
Average ABG				
Ph	7.20±0.03	7.39±0.02	<0.0001	26.28
PaCO₂	55.73±3.40	40.01±1.84	<0.0001	20.55
PaO₂	54.86±3.07	80.42±4.22	<0.0001	24.48
P/F ratio	68.40±3.86	160.61±8.32	<0.0001	48.66
Lactate	3.70±0.61	1.73±0.21	<0.0001	14.76

Non-survivors demonstrated significantly worse ventilatory mechanics both on day 1 and during ICU stay. On day 1, respiratory rate, peak inspiratory pressure (PIP), plateau pressure (PP), and driving pressure (DP) were significantly higher in non-survivors, while static compliance (SC) and dynamic compliance (DC) were significantly lower (all p<0.0001). Total mechanical power (TMP), mechanical power dynamic (MPD), and mechanical power resistive (MPR) were also significantly elevated in the non-survivor group. Average ventilatory parameters showed persistent derangements among non-survivors, including significantly higher respiratory rate, PIP, PEEP, PP, DP, mechanical power static (MPS), MPD, and TMP, along with significantly reduced static and dynamic compliance (Table 3).

**Table 3:** Ventilator and mechanical power parameters Data are presented as mean±SD. Comparisons between survivors and non- survivors were performed using Student’s t-test. Statistical significance was defined as p<0.05. PIP: peak inspiratory pressure, PEEP: Positive end-expiratory pressure, SC: static compliance, DC: dynamic compliance, PP: plateau pressure, DP: driving pressure, MPS: mechanical power static, MPD: mechanical power dynamic, MPR: mechanical power resistive, TMP: total mechanical power, SD: standard deviation

Parameters	Non-survivor	Survivor	P-value	Test statistic
Day 1 parameters				
Respiratory rate	19.56±1.68	16.93±0.89	<0.0001	6.9
Tidal volume	358.85±26.32	385.93±12.06	<0.0001	4.72
PIP	37.47±2.75	32.95±2.40	<0.0001	6.21
PEEP	9.93±0.85	9.98±0.62	0.8001	0.24
SC	20.79±3.35	24.77±1.92	<0.0001	5.18
DC	17.28±3.20	21.06±1.86	<0.0001	5.16
PP	29.20±1.60	26.89±1.19	<0.0001	5.84
DP	19.27±1.51	17±1.89	<0.0001	4.7
MPS	6.84±1.10	6.38±0.35	0.07	1.97
MPD	6.62±0.89	5.67±1.41	<0.0001	2.84
MPR	5.63±1.58	3.85±1.09	<0.0001	4.67
TMP	19.09±2.68	15.90±2.12	<0.0001	4.65
Average parameters				
Respiratory rate	22.04±3.87	18.16±3.56	<0.0001	3.7
Tidal volume	445.96±59.19	434±46.63	0.40	0.79
PIP	37.46±3.55	30.24±3.81	<0.0001	6.95
PEEP	11.26±3.13	8.33±2.22	0.002	3.82
SC	23.60±5.30	30.57±9.46	0.004	3.25
DC	17.32±3.15	20.68±5.08	0.01	2.84
PP	30.90±3.80	23.55±2.84	<0.0001	7.71
DP	19.64±4.29	15.22±3.89	0.002	3.82
MPS	20.27±5.04	12.25±2.89	<0.0001	6.86
MPD	26.63±7.03	17.72±5.75	<0.0001	4.9
MPR	6.36±4.09	5.47±3.95	0.30	0.78
TMP	26.63±7.03	17.72±5.75	<0.0001	4.9

Quantitative analysis of lung aeration based on chest CT demonstrated no statistically significant differences between survivors and non-survivors in well-aerated, poorly aerated, non-aerated, or hyperinflated lung volumes (p>0.05 for all parameters) (Table 4).

**Table 4:** Quantitative lung aeration parameters Data are presented as mean±SD (mL). Comparisons between groups were performed using Student’s t-test. Statistical significance was defined as p<0.05. SD: standard deviation

Lung volume	Non-survivor	Survivor	P-value	Test statistic
Well aerated (mL)	822±156.69	817.12±134.94	0.9011	0.12
Poorly aerated (mL)	609.40±161.95	636.24±156.14	0.5521	0.6
Non aerated (mL)	648.04±91.46	626.28±85.90	0.4512	0.87
Hyperinflated (mL)	57.08±33.38	47.48±28.91	0.3003	1.09

Univariate analysis identified several significant predictors of mortality. APACHE II score (rho=0.6175, p<0.0001) and SOFA score (rho=0.6094, p<0.0001) were strongly associated with mortality. Day 1 and average ABG parameters, including lower pH, lower PaO₂, lower P/F ratio, and higher PaCO₂ and lactate levels, showed strong correlations with mortality (all p<0.0001). Among ventilatory parameters, higher respiratory rate, PIP, PP, DP, and mechanical power indices were significantly associated with mortality, whereas reduced tidal volume, static compliance, and dynamic compliance were inversely associated with survival. Average ventilatory parameters similarly demonstrated strong associations between elevated ventilatory pressures, increased mechanical power, and mortality. CT-derived aeration parameters did not significantly correlate with mortality (Table 5).

**Table 5:** Univariate analysis of predictors of mortality Results are presented as Spearman’s rho correlation coefficients with 95% confidence intervals and p-values. Statistical significance was defined as p<0.05. APACHE II: Acute Physiology and Chronic Health Evaluation II, SOFA: Sequential Organ Failure Assessment, ABG: arterial blood gas, PaCO₂: partial pressure of carbon dioxide, PaO₂: partial pressure of oxygen, P/F ratio: PaO₂/FiO₂ ratio, PIP: peak inspiratory pressure, PEEP: positive end-expiratory pressure, SC: static compliance, DC: dynamic compliance, PP: plateau pressure, DP: driving pressure, MPS: mechanical power static, MPD: mechanical power dynamic, MPR: mechanical power resistive, TMP: total mechanical power, CI: confidence interval

Predictors of mortality	Rho	95% CI	P-value
Clinical parameters			
APACHE II score	0.6175	0.4026 to 0.7680	<0.0001
SOFA score	0.6094	0.3915 to 0.7625	<0.0001
Day 1 ABG			
pH (day 1)	-0.868	-0.9242 to -0.7733	<0.0001
PaCO₂ (day 1)	0.8579	0.7578 to 0.9186	<0.0001
PaO₂ (day 1)	-0.861	-0.9202 to -0.7623	<0.0001
P/F ratio (day 1)	-0.866	-0.9235 to -0.7712	<0.0001
Lactate (day 1)	0.8597	0.7607 to 0.9196	<0.0001
Average ABG			
pH (average)	-0.87	-0.9258 to -0.7777	<0.0001
PaCO₂ (average)	0.8662	0.7712 to 0.9235	<0.0001
PaO₂ (average)	-0.866	-0.9235 to -0.7712	<0.0001
P/F ratio (average)	-0.866	-0.9235 to -0.7712	<0.0001
Lactate (average)	0.8663	0.7713 to 0.9235	<0.0001
Day 1 ventilator			
Respiratory rate (day 1)	0.7671	0.6163 to 0.8637	<0.0001
Tidal volume (day 1)	-0.712	-0.8291 to -0.5346	<0.0001
PIP (day 1)	0.7114	0.5339 to 0.8288	<0.0001
PEEP (day 1)	-0.098	-0.3733 to 0.1940	0.5002
SC (day 1)	-0.62	-0.7696 to -0.4059	<0.0001
DC (day 1)	-0.676	-0.8061 to -0.4832	<0.0001
PP (day 1)	0.6371	0.4293 to 0.7809	<0.0001
DP (day 1)	0.6077	0.3893 to 0.7614	<0.0001
MPS (day 1)	0.305	0.02063 to 0.5436	0.0313
MPD (day 1)	0.5989	0.3774 to 0.7555	<0.0001
MPR (day 1)	0.5807	0.3533 to 0.7433	<0.0001
TMP (day 1)	0.6292	0.4184 to 0.7757	<0.0001
Average ventilator			
Respiratory rate (average)	0.4758	0.2196 to 0.6706	0.0005
Tidal volume (average)	0.1053	-0.1864 to 0.3800	0.4665
PIP (average)	0.7471	0.5863 to 0.8513	<0.0001
PEEP (average)	0.4686	0.2107 to 0.6655	0.0006
SC (average)	-0.427	-0.6355 to -0.1604	0.002
DC (average)	-0.343	-0.5725 to -0.06246	0.0149
PP (average)	0.7915	0.6533 to 0.8786	<0.0001
DP (average)	0.4825	0.2278 to 0.6754	0.0004
MPS (average)	0.7359	0.5698 to 0.8443	<0.0001
MPD (average)	0.5724	0.3423 to 0.7377	<0.0001
MPR (average)	0.1081	-0.1837 to 0.3824	0.4549
TMP (average)	0.5724	0.3423 to 0.7377	<0.0001
Investigations			
Well aerated (mL)	-0.04	-0.3226 to 0.2488	0.7817
Poorly aerated (mL)	-0.094	-0.3704 to 0.1972	0.515
Non aerated (mL)	0.1289	-0.1632 to 0.4003	0.3723
Hyperinflated (mL)	0.1442	-0.1481 to 0.4133	0.3178

Multivariate regression analysis identified independent predictors of mortality in patients with ARDS. Average PaO₂ (B=0.028, p<0.0001) and average P/F ratio (B=-0.016, p<0.0001) emerged as strong independent predictors. Among ventilatory parameters, day 1 static compliance (B=-0.018, p=0.015), plateau pressure (B=0.061, p=0.033), and mechanical power static (B=-0.072, p=0.032) remained independently associated with mortality. Additionally, average PIP (B=-0.017, p=0.049) and average total mechanical power (B=0.012, p=0.037) were significant predictors of adverse outcomes (Table 6).

**Table 6:** Multivariate regression for predictors of mortality Results are presented as unstandardized coefficients (B), standardized coefficients (beta), 95% confidence interval, and p-values. Logistic regression analysis was performed. Statistical significance was defined as p<0.05. APACHE II: Acute Physiology and Chronic Health Evaluation II, SOFA: Sequential Organ Failure Assessment, ABG: arterial blood gas, PaCO2₂: partial pressure of carbon dioxide, PaO₂: partial pressure of oxygen, P/F ratio: PaO₂/FiO₂ ratio, PIP: peak inspiratory pressure, PEEP: positive end-expiratory pressure, SC: static compliance, DC: dynamic compliance, PP: plateau pressure, DP: driving pressure, MPS: mechanical power static, MPD: mechanical power dynamic, MPR: mechanical power resistive, TMP: total mechanical power

Predictors	B (unstandardized)	Beta (standardized)	P-value	95% lower	95% upper
Constant	6.558		0.005	2.206	10.909
APACHE II score	0.001	0.024	0.218	-0.001	0.004
SOFA score	-0.002	-0.013	0.455	-0.006	0.003
pH (day 1)	-0.166	-0.031	0.5	-0.673	0.34
PaCO₂ (day 1)	0.002	0.028	0.222	-0.001	0.004
PaO₂ (day 1)	0.003	0.075	0.475	-0.005	0.01
P/F ratio (day 1)	-0.001	-0.106	0.543	-0.005	0.003
Lactate (day 1)	-0.032	-0.065	0.157	-0.078	0.013
pH (average)	-0.578	-0.11	0.153	-1.391	0.235
PaCO₂ (average)	0.002	0.03	0.453	-0.003	0.007
PaO₂ (average)	0.028	0.737	<0.0001	0.018	0.037
P/F ratio (average)	-0.016	-1.478	<0.0001	-0.02	-0.011
Lactate (average)	0.025	0.055	0.35	-0.03	0.081
Respiratory rate (day 1)	0.015	0.056	0.43	-0.024	0.054
Tidal volume (day 1)	0.001	0.045	0.324	-0.001	0.003
PIP (day 1)	-0.044	-0.3	0.129	-0.103	0.014
SC (day 1)	-0.018	-0.123	0.015	-0.033	-0.004
DC (day 1)	0.005	0.031	0.464	-0.009	0.019
PP (day 1)	0.061	0.218	0.033	0.005	0.116
DP (day 1)	-0.035	-0.141	0.103	-0.078	0.008
MPS (day 1)	-0.072	-0.12	0.032	-0.138	-0.007
MPD (day 1)	-0.002	-0.005	0.81	-0.018	0.014
MPR (day 1)	0.07	0.225	0.114	-0.018	0.159
Respiratory rate (average)	-0.008	-0.07	0.188	-0.021	0.004
PIP (average)	-0.017	-0.173	0.049	-0.034	0
PEEP (average)	0.008	0.049	0.278	-0.007	0.023
SC (average)	-0.001	-0.02	0.401	-0.004	0.002
DC (average)	-0.007	-0.06	0.198	-0.017	0.004
DP (average)	0.004	0.038	0.551	-0.01	0.018
MPS (average)	-0.003	-0.036	0.699	-0.02	0.014
TMP (average)	0.012	0.185	0.037	0.001	0.023

Overall, the findings demonstrate that impaired oxygenation and adverse ventilatory mechanics, particularly elevated pressures and increased mechanical power, were major determinants of mortality among patients with ARDS requiring mechanical ventilation.

## Discussion

Mechanical ventilation stands as a life-saving intervention in the management of respiratory failure. However, it carries the risk of ventilator-induced lung injury. Despite the adoption of lung-protective ventilation strategies, mortality rates remain high. The concept of mechanical power has emerged as a comprehensive metric encompassing key ventilator parameters associated with the genesis of ventilator-induced lung injury, including volume, pressure, flow, resistance, and respiratory rate [12]. In this context, the reduction of mechanical power, that is, the total energy released into the lung per unit time, derived from the interaction between the ventilator setting and lung conditions, could be considered an alternative approach to minimize VILI [11].

In our study, baseline demographic variables such as age, gender, and BMI did not differ significantly between survivors and non-survivors, suggesting that disease severity and physiological derangements were more important determinants of outcome than demographic characteristics alone. In contrast, APACHE II and SOFA scores were significantly higher among non-survivors and demonstrated strong positive correlations with mortality on univariate analysis.

Sepsis and pulmonary infections were the predominant etiological factors, consistent with existing literature identifying pneumonia and systemic infection as major contributors to ARDS development [13].

Arterial blood gas analysis revealed markedly impaired oxygenation and ventilation among non-survivors. Lower pH, lower PaO₂, lower P/F ratio, and elevated PaCO₂ and lactate levels were consistently associated with mortality both on day 1 and throughout ICU stay. Persistent hypoxemia and hypercapnia likely reflect severe alveolar injury, impaired gas exchange, and progressive respiratory failure. The strong correlations observed between average oxygenation parameters and mortality emphasize the importance of sustained oxygenation status rather than isolated measurements alone. Similar observations were reported by Coppola et al., who demonstrated significantly lower PaO₂/FiO₂ ratios and higher PaCO₂ levels among non-survivors [11].

Ventilatory mechanics demonstrated significant differences between survivors and non-survivors. Non-survivors had significantly higher respiratory rates, peak inspiratory pressures, plateau pressures, and driving pressures, along with lower static and dynamic compliance. These findings support the concept that increased mechanical stress and reduced lung compliance contribute substantially to adverse outcomes in ARDS. Elevated plateau pressure and driving pressure have previously been associated with alveolar overdistension and progression of VILI, thereby worsening mortality risk [5]. Mechanical power parameters also differed significantly between the two groups. Total mechanical power, mechanical power dynamic, and mechanical power resistive were higher among non-survivors, suggesting that increased energy delivery to injured lungs may contribute to ongoing pulmonary damage. Although mechanical power static did not differ significantly on day 1, average mechanical power indices during ICU stay remained significantly elevated among non-survivors. These findings indicate that cumulative ventilatory energy exposure over time may have greater prognostic relevance than isolated early measurements.

The univariate analysis further demonstrated that multiple ventilatory variables, including respiratory rate, plateau pressure, driving pressure, and mechanical power indices, were significantly associated with mortality. Conversely, reduced static and dynamic compliance were inversely associated with survival. These observations reinforce the hypothesis that impaired respiratory mechanics and excessive ventilatory stress are central contributors to poor outcomes in patients with ARDS receiving invasive mechanical ventilation [10].

Interestingly, quantitative CT-derived aeration parameters did not demonstrate significant associations with mortality. Well-aerated, poorly aerated, non-aerated, and hyperinflated lung volumes were comparable between survivors and non-survivors. This suggests that static radiological assessment of aeration alone may not adequately capture the dynamic physiological and mechanical factors influencing outcome in ARDS.

Overall predictors for mortality: independent predictors

Multivariate regression analysis identified average PaO₂, average P/F ratio, day 1 static compliance, plateau pressure, mechanical power static, average peak inspiratory pressure, and average total mechanical power as independent predictors of mortality. These findings highlight the combined importance of oxygenation efficiency and ventilatory mechanics in determining outcomes among patients with ARDS. In particular, the independent association of total mechanical power with mortality supports the growing evidence that cumulative ventilatory energy may play a clinically significant role in VILI pathogenesis [10].

Our findings are partially consistent with previous investigations evaluating mechanical power in ARDS. Coppola et al. reported that mechanical power normalized to compliance or well-aerated lung tissue, rather than absolute mechanical power alone, independently predicted ICU mortality [11]. Similarly, Khorasanee et al. demonstrated improved mortality prediction when mechanical power was normalized to respiratory system compliance [14]. In contrast, Medhi et al. observed that driving pressure, rather than mechanical power itself, independently predicted mortality after adjustment for confounding variables [15]. These differences may reflect variations in study population, ventilatory strategies, ARDS severity, and methods used for mechanical power calculation.

The present study has important clinical implications. The findings suggest that optimization of ventilatory mechanics should extend beyond conventional low tidal volume strategies and include careful monitoring of plateau pressure, compliance, and mechanical power. Reducing cumulative mechanical energy transfer to the lungs may potentially minimize ventilator-induced injury and improve survival outcomes. Continuous assessment of oxygenation indices and respiratory mechanics may therefore help guide individualized ventilatory strategies in critically ill patients with ARDS.

The study has several limitations. The relatively small sample size and single-center design may limit the generalizability of the findings. Since it was an observational study, we were not able to match the baseline difference in disease severity, which may have confounded the association. Mechanical power calculations were derived using bedside ventilatory parameters and may not fully account for regional lung heterogeneity. Additionally, serial changes in ventilatory management and adjunctive therapies were not separately analyzed. Larger multicentric studies are required to validate the prognostic utility of mechanical power and establish clinically relevant threshold values for bedside application.

## Conclusions

In this 12-month prospective study of mechanically ventilated patients with ARDS admitted to a tertiary ICU, mortality was driven primarily by the severity of physiological derangements rather than baseline demographic factors. While age, sex, and BMI were not associated with outcomes, non-survivors consistently exhibited higher disease severity scores, profound abnormalities in oxygenation and acid-base status, and markedly impaired ventilatory mechanics from the onset of ICU care. These disturbances, particularly severe hypoxemia, lactic acidosis, elevated ventilatory pressures, reduced compliance, and increased mechanical power, showed strong and consistent correlations with mortality, underscoring their clinical relevance. Multivariate analysis further revealed that oxygenation indices (average PaO₂ and P/F ratio) and ventilatory stress markers (day 1 static compliance, plateau pressure, and mechanical power; average PIP and total mechanical power) were the strongest independent predictors of death. In contrast, CT-derived aeration parameters did not predict outcomes. Overall, the findings highlight that both the degree of oxygenation impairment and the mechanical load imposed by ventilation, especially early in the course of illness, play decisive roles in determining survival. These insights emphasize the importance of optimizing ventilatory strategies and maintaining lung-protective parameters to improve outcomes in patients with ARDS.
